# Conflict and HIV: A framework for risk assessment to prevent HIV in conflict-affected settings in Africa

**DOI:** 10.1186/1742-7622-1-6

**Published:** 2004-10-29

**Authors:** Nancy B Mock, Sambe Duale, Lisanne F Brown, Ellen Mathys, Heather C O'Maonaigh, Nina KL Abul-Husn, Sterling Elliott

**Affiliations:** 1 Tulane University Center for International Resource Development, New Orleans, United States; 2 Department of International Health and Development, Tulane University School of Public Health and Tropical Medicine, New Orleans, United States

## Abstract

In sub-Saharan Africa, HIV/AIDS and violent conflict interact to shape population health and development in dramatic ways. HIV/AIDS can create conditions conducive to conflict. Conflict can affect the epidemiology of HIV/AIDS. Conflict is generally understood to accelerate HIV transmission, but this view is simplistic and disregards complex interrelationships between factors that can inhibit and accelerate the spread of HIV in conflict and post conflict settings, respectively. This paper provides a framework for understanding these factors and discusses their implications for policy formulation and program planning in conflict-affected settings.

## Introduction

Of the obstacles to development in the sub-Saharan African (SSA) region, perhaps none has had a more profound impact than the dual burdens of HIV/AIDS and conflict. During the past quarter century, no region of the world has been more acutely affected by large-scale violent conflict than SSA. Almost all SSA countries have directly experienced, or border a country that has directly experienced, violent conflict. The number of states engaged in significant violent conflicts doubled between 1989 and 2000, from 11 to 22 [1,2]. A "conflict belt" stretches from Angola to the Horn of Africa, while peace in western Africa remains elusive.

The same quarter century has seen the development of an even more pervasive crisis – the HIV/AIDS pandemic. SSA continues to account for the large majority (66%) of the world's HIV/AIDS cases [3], even though its population comprises only 10% of the world's total population. There is great diversity across Africa in the levels and trends of HIV infection. While stabilization of the epidemic appears to have begun in several countries, population prevalence rates across most of the continent continue to increase, including among rural populations and sociodemographic groups not previously considered at elevated risk.

Although much has been written about HIV and conflict individually, surprisingly little has been written about the dynamics of the relationship between the two crises. In fact, an extensive keyword search of the internet and peer-reviewed journal databases turned up fewer than 100 references to the intersection of HIV and conflict. Virtually all studies are descriptive and only one utilized hypothesis-testing analytical strategies [4]. Although constraints on conducting such research are considerable, research on the interface between the two crises is of critical importance. A clearer understanding of the dynamics of the interface between conflict and HIV is crucial for the development of effective and efficient strategies to reduce population risk.

Some literature on conflict-affected countries in SSA underscores mechanisms by which HIV/AIDS and violent conflict may exhibit bi-directional causal associations on the population level. HIV/AIDS has been recognized to play a potential role in creating conditions conducive to violent conflict on the continent, although there is as yet no good empirical evidence to that effect. A policy forum held at the United States Institute for Peace [2] concluded that the pandemic will emerge as a deeply destabilizing force across the social, political, and economic landscapes of Africa. For example, large numbers of AIDS orphans and vulnerable children strain the social support networks in many southern African countries [5,6]. The psychological trauma and the lack of parental affection and supervision experienced by AIDS orphans put them at risk of involvement with criminal or antisocial activities (e.g. as child soldiers), or activities that serve to further their risk of contracting and further propagating the disease (e.g. as child prostitutes). The extent of the risks faced by AIDS orphans – e.g. risks for anti-social and criminal behavior later in life – needs to be sufficiently researched. By causing elevated AIDS-related mortality among the 15–45 year-old age group, the illness degrades human capital, undermines household capacity and stability, reduces economic productivity, and robs social and political institutions of intellectual resources [7].

On the other hand, violent conflict clearly influences the epidemiology of HIV. To date, the literature emphasizes conflict as a risk factor for HIV transmission. Conflict destroys social and physical infrastructure, resulting in untreated sexually transmitted infections (STIs), poor health and malnutrition and, as a consequence, increased risk of transmission in the event of viral exposure. The migration and poverty created or exacerbated by conflict may result in increased exposure opportunity through: (1) increased prevalence of casual or commercial sexual activity; (2) increased interactions among civilians and combatants/military personnel, known for their high risk behaviors; (3) the development of cultures of violence that promote sexual violence and predation; (4) mass migration, which increases sexual mixing among populations; and (5) the destruction of public health education mechanisms (e.g. mass media, health facilities, and formal education), which negatively affects public health-related knowledge, attitudes, and practices [8,9].

**Figure 3:**
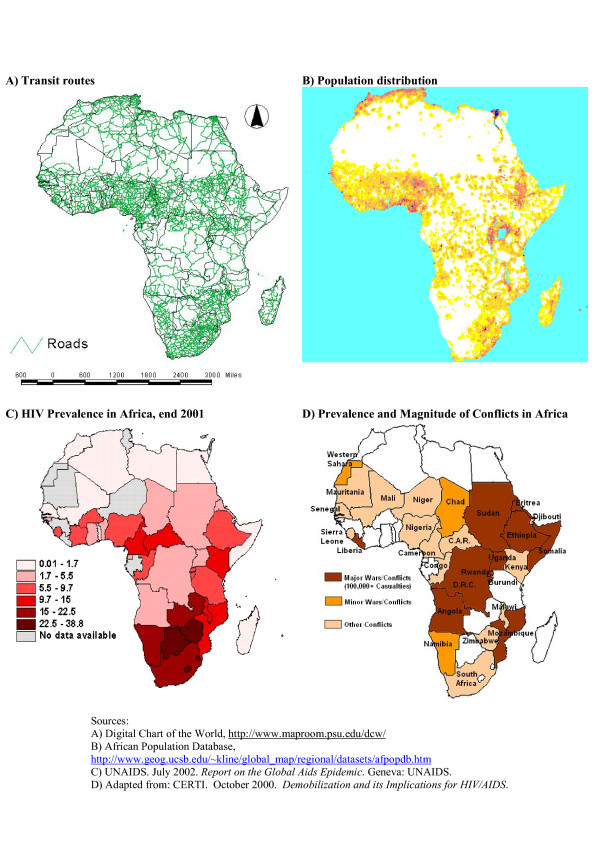
Maps of Africa

However, the epidemiologic data despite their limitations^2^, suggest that the relationship between conflict and HIV levels may be more complex than this general picture of joint potentiation implies. With few exceptions, countries that have experienced widespread violent conflict have apparently lower levels of HIV infection compared to those that have experienced relative peace (compare figures 3c and 3d). While many factors operate during conflict to increase vulnerability of affected populations to HIV, exposure opportunities at an aggregate level may actually be lower, therefore decreasing HIV risk. Alternatively, when conflict subsides, exposure opportunity may increase, leading to the potentially explosive spread of HIV. At the end of the Angola conflict in 2002, the country HIV prevalence was relatively lower than the rates in other Southern African countries, which suggests that conflict may have slowed HIV spread in this case [10].

The present paper evolved out of the observation that available evidence for conflict-affected countries in SSA suggests a complex set of possible net effects of conflict on the HIV epidemic, with multiple and quite variable outcomes. The implications of the analysis and findings have great significance for developing policies and programs to confront HIV in the context of conflict. These are discussed along with recommendations for improved policies and programs to address this important constraint to African health development.

## A Framework for Understanding the Interrelationships between Conflict and HIV

Our framework develops two important aspects of the problem of HIV and conflict in SSA. These are:

• the importance of ecological in addition to individual risk factor explanatory models, and

• the importance of comparing the influences of conflict both during and post conflict.

We also develop the notion that conflict is a complex social phenomenon and that its effects are highly contextualized.

To articulate a framework for understanding the interface between the two crises, the concepts of *vulnerability*, *hazard exposure opportunity *and *risk *are useful. These are borrowed from the disaster literature [11] but are germane for understanding the evolution of HIV/AIDS as a crisis. Vulnerability is the ability of a population to withstand hazards or shocks to the system when they are present. Classical vulnerability factors include poverty, low education/knowledge, poor social infrastructure and attitudinal factors. Additional factors specific to conflict and HIV include levels of economically-motivated sex, levels and types of civil-military interactions, migration patterns, sex-related knowledge and attitudes, and population health status (particularly STIs and nutrition). Vulnerable groups in conflict settings may include child-headed households, child soldiers, unaccompanied children, women, demobilized soldiers, and repatriating refugees. Vulnerability means that, when exposed to a hazard, an individual or group is more likely to experience adverse effects (risk).

Hazards or shocks to human communities such as conflict affect the HIV exposure opportunity of members of these affected communities. HIV exposure opportunity can be mediated by war or other ways in which these communities interact with other communities (regional human ecology), which ultimately affects disassortive mixing (i.e. mixing of population groups of differing levels of HIV prevalence). In Southern Africa, the regional ecology is one that favors extraordinarily high HIV risk through, for example, high population density, good physical infrastructure, economically-motivated mobility, high poverty levels, and large wealth disparities within and between countries. By lowering exposure opportunity, war may lead to isolation from the general regional ecology and, therefore, to lower HIV risk at the population level. Alternatively, war leads to a changed and often more intense mixing of mobile military populations and civilians, which can increase HIV risk through disassortive mixing.

**Figure 1:**
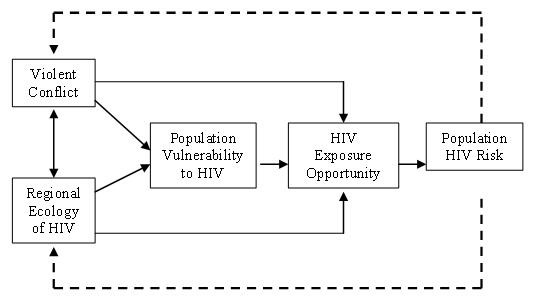
Conceptual Framework of Principal Causes of HIV Risk in Conflict-Affected Populations

Figure 1 illustrates how conflict shocks may affect vulnerability and exposure opportunity to affect population HIV risk. Conflict shocks can weaken a community's ability to avoid HIV exposure/infection (vulnerability), or it can influence exposure opportunity itself. Vulnerability and exposure are the basic determinants of population-level HIV risk. Underlying determinants here include violent conflict and the regional ecology of HIV, which exhibit measurable effects on both vulnerability and exposure opportunity (and interact with each other). Finally, HIV infection goes on to influence the progression of conflict and the social ecology of HIV in a feedback loop. The model has utility for structured inquiry into the effects of conflict on HIV. It draws attention to mechanisms by which vulnerability and risk of exposure, and therefore risk of transmission, may be increased *or *decreased during conflict. The relative importance of each of the components, and therefore the ultimate effect of conflict (i.e. augmenting or reducing risk), will be highly context specific.

**Figure 2:**
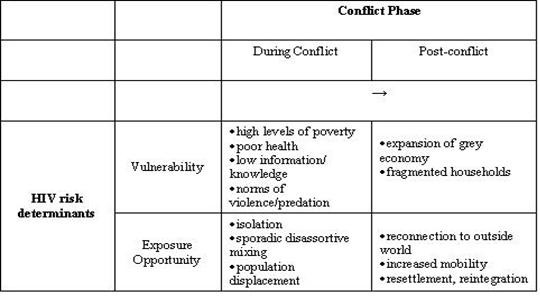
HIV Vulnerability and Exposure Opportunity in Relation to Conflict Phase

Figure 2 illustrates that there is an important time dimension to consider as well. The most basic distinction of interest is that of conflict and post conflict, although these are rarely clear-cut distinctions. What is important to note here is that vulnerability increases during conflict and rarely decreases rapidly after conflict subsides because of the profound societal changes that generally occur during conflict. On the other hand, opportunity for exposure to HIV may change dramatically post conflict due to decreased population isolation and rapid improvement in freedom of movement. These changes will likely be most acute in countries with high economic potential, such as Angola, Côte d'Ivoire and the Democratic Republic of Congo. The juxtaposition of high vulnerability and increased exposure opportunities post-conflict can lead to explosive growth in the epidemic. This already has occurred to some extent in Mozambique [12].

This finding is critical in that it explains in part why, in some chronic conflict settings, apathy has developed towards HIV because seroprevalence levels may be low. However, the potential for rapid progression of the infection post conflict due to high vulnerability argues for a much more aggressive and deliberate approach to HIV in post-conflict settings.

## Conflict as an Analytical Factor

Considerable research has been conducted to date to construct comprehensive typologies of conflict. Several well-established research initiatives, such as the Correlates of War Project [13], have developed typologies of conflict for application to conflict early warning and prevention efforts. These typologies categorize conflicts based upon variables associated with either the *determinants *of conflict or the *manifestations *of conflict. Typologies that focus on the determinants of conflict categorize conflicts primarily based upon characteristics of the political, legal, economic, social, or cultural environments that are associated with greater risk of conflict. Examples of such factors include [14]:

• political/legal status and structure of warring parties (e.g. state, non-state, internal, or external; military capabilities and levels of discipline of combatants);

• economic context (e.g. economic disparities, distribution of poverty, and access to critical resources);

• nature of political system (e.g. democratic or authoritarian);

• self-identified characteristics distinguishing warring parties (e.g. ethnic, religious, class, regional, or social identity);

• characteristics of physical environment as it affects the conflict (e.g. mountainous or flat); and

• purpose of conflict (e.g. motives of combatants, points of contention, and ideological differences).

In contrast, some approaches focus on the manifestations of conflict, defining categories in terms of measurable aspects of the conflict and its effects on populations. Examples include:

• geographic scale of conflict (e.g. sub national, national, or international; inter-state, extra-state, or intra-state);

• time-scale of conflict (e.g. duration and onset);

• types of military technology, tactics, and funding methods employed;

• involvement of civilians;

• levels of mortality (e.g. battle-related and indirect);

• levels of other negative health outcomes (e.g. morbidity and malnutrition); and

• levels of displacement (e.g. internal or cross-border).

A determinant-based typology has more direct application to the critical areas of conflict early warning and prevention, despite the analytical complexities derived from the high degree of interdependence (i.e. interaction) among these determinants [15]. However, most existing classification schemes fail to incorporate an emphasis on the public health impacts of conflicts other than battle-related fatalities. For example, other categories of war-related mortality, such as civilian deaths, deaths following famine or disease and deaths that are allowed to occur for political reasons, are often omitted. Similarly, morbidity, malnutrition and displacement among civilians, highly characteristic of post-Cold War conflicts in SSA, have been largely overlooked [14]. These population-level results of conflict appear, in many cases, to help explain the links between conflict and HIV/AIDS progression. Therefore, the present framework draws from both types of typologies for maximum utility regarding the problem of HIV and conflict, recognizing the complex nature of conflict as an epidemiologic study factor.

## Aspects of Violent Conflict that Directly Affect the HIV Epidemic

Despite large cross-cultural and historical variation in the determinants and manifestations of conflict, and variation in the systems that have been developed to analyze conflict, a model can be developed specific to inquiry about the public health impacts of conflict on HIV epidemiology. Conflict can be analyzed in terms of the following dimensions, all of which may help to shape its influence on HIV risk.

### Time-scale of conflict

Both the *duration *of the conflict and the *point of conflict onset *relative to the progression of the epidemic should be considered. Chronic conflict, for example, may engender profound societal changes that are manifested in immediate increases in population risk of HIV. For example, widespread impoverishment resulting from war may fuel high levels of commercial sex as a survival strategy. Similarly, displaced populations are believed to be at greater risk of sexual violence or sexual activity as a way to negotiate access to key resources for survival. A recent finding regarding the behavior of humanitarian aid workers in western Africa tragically supports this assertion [16].

Longer-duration conflicts generally lead to greater cumulative effects of conflict on social infrastructure. Populations may be isolated from modern communications for a long period of time, resulting in much greater naïveté regarding the epidemiology and prevention of the disease. In low-prevalence conflict settings this may not impact transmission significantly; post conflict, however, this may exacerbate vulnerability.

Alternatively, chronic conflict may result in lower exposure opportunities following reduced social mixing due to isolation and limited population mobility. During the war in Angola, for example, mobility was limited and most of the population was concentrated in small "islands of security" around provincial capitals. HIV seroprevalence remained low as a result.

Equally, the point of conflict onset relative to the progression of the epidemic may determine the degree to which conflict may result in sustained low seroprevalence levels for the duration of the war. In Rwanda, for example, sentinel surveys among pregnant women and STI patients demonstrated prevalence rates exceeding 30% before the 1994 genocide [17]. Because HIV was already so prevalent, community isolation did not likely curtail the local spread of the disease. In contrast, prevalence rates in Mozambique in sentinel surveys of high-risk populations appear to have remained relatively low until the war ended, at which point the resumption of normal patterns of social mixing occurred alongside a marked rise in prevalence rates [17]. Thus, when initial low prevalence rates are combined with isolation for long periods of time, HIV progression at the population level may be considerably attenuated.

### Characteristics and involvement of parties involved in conflict

The *political/legal status*, *differential HIV prevalence rates*, *relative size*, *motives*, and *tactics *characterizing combatants and affected civilian populations may all shape the progression of the epidemic. In terms of political/legal status, combatant groups may comprise government military forces, paramilitary forces, organized rebel groups, or highly fragmented rebel groups. Available data suggest but do not conclusively confirm the oft cited axiom that significant differentials in seroprevalence can exist between government military and civilian populations in Africa [18]. More recent findings suggest that this too is contextualized [19]. Almost no data is available regarding other types of armed groups.

Modern conflict in SSA often inflicts a high degree of sexual violence upon civilian populations that, if civil-military seroprevalence differentials exist, will result in increased mixing and HIV spread. Additionally, commercial sexual activity and other commercial activities may follow soldiers, again facilitating HIV spread. The size and motives/tactics of armed groups may determine in part the types of interactions (and violence of those interactions) with the civilian population.

### Geographic scale and dynamics of conflict

The *scale *and *geographic focus *of the conflict highlight geographic areas that may be differentially affected by the conflict or the epidemic. In terms of scale, conflicts may take place on a regional, national, or sub-national level. Most post-Cold War conflicts in SSA involve civil conflict; many conflicts have international and regional dimensions. The war that started in the Democratic Republic of Congo (DRC) in 1998 drew armies from at least seven African countries experiencing wide variations in their levels of HIV prevalence. The concept of the geographic focus of the conflict highlights the fact that the effects of war are unlikely to be homogeneous in any situation. This gives rise to distinct local ecologies that may experience lower HIV vulnerability and exposure to HIV hazards through the factors listed above, particularly isolation and population mixing associated with military and forced migration. For example, the Ethiopian conflict resulted in a large concentration of military along the Ethiopian-Eritrean border and accompanying civilian commercial activity. In some cases, such as Mozambique, conflicts have resulted in intensified and highly localized trading corridors between outside countries of higher seroprevalence and lower prevalence areas.

## Specific Mechanisms through which Conflict Influences HIV Risk

### Factors that May Decrease Risk

Factors may decrease risk by decreasing vulnerability, or more commonly, by decreasing exposure opportunity. The following factors are postulated to accompany conflict in post-Cold War SSA and may explain how conflict may actually limit the spread of the epidemic during the period of conflict.

#### Factor 1: Increased isolation of communities

Conflict isolates communities by destroying transport systems, making travel unsafe, and disrupting the market-based activities that encourage economic migration. Conflict may freeze normal cross-border migration, as in the case of Ethiopia and Eritrea. On the sub-national level, civil conflict may isolate rural communities controlled by armed factions, as in Angola, or limit population movement because of the loss of transport infrastructure, as in the Democratic Republic of Congo. Where conflict is chronic, the effect of limiting population movement and therefore population mixing could be a very significant contributor to the relatively lower levels of HIV found among affected populations.

#### Factor 2: Increased death rates among high risk groups

Another aspect of conflict that may decrease population HIV risk is differential mortality among high-risk groups. It is well established that mortality among adult males is elevated in conflict-affected settings. Post-genocide Rwanda has shown a demographic shift such that among adults between the ages of 15 and 54 years, women far outnumber men [20]. In addition, poor nutrition and the lack of access to health services probably decreases the survival time of HIV-infected individuals. A recent study in Guinea Bissau demonstrated a strong differential in mortality between war-affected cohorts of a tuberculosis treatment program according to HIV status [21].

#### Factor 3: Decreased casual sex associated with trauma and depression

Available evidence points to the unexplored possibility that conflict may result in decreased sexual activity, most commonly as a result of psychological sequelae of trauma such as post-traumatic stress disorder (PTSD). It has been shown that absent or low libido in those with post-traumatic stress disorder can be as high as 69% [22]. In those patients with general depression, reduced libido is present in up to three quarters of patients [23]. An elevated level of depression in conflict-affected settings has been documented [24]. Research in Rwanda in the late 1990s investigated the prevalence of clinical depression (as per the Diagnostic and Statistical Manual for Mental Disorders, DSM-IV criteria, measured using an adapted version of the Hopkins Symptom Checklist); the prevalence of a local severe depression-like syndrome of "mental trauma" *(guhahamuka)*; and the prevalence of a local, less acute syndrome of "severe grief" *(agahinda gakabije)*. In the commune that experienced significantly lower levels of violence during the genocide (Butamwa), only 5.6% of adults had depression, and 31.9% of adults had *agahinda gakabije*. In the commune that experienced widespread violent conflict during the genocide, almost one-fifth (17.9%) of adults had depression and almost half (41.8%) of adults had *agahinda gakabije *[24]. The results, though inconclusive, suggest that depression-like syndromes may be highly prevalent in some settings.

#### Factor 4: Disruption of sexual networks following conscription or displacement

Conflict may lead to the disruption of sexual networks associated with forced migration and/or the conscription of husbands and sexual partners. Females may have fewer opportunities for casual sex, though this factor remains unexplored in the research literature. This may hold most true in displaced communities, which are often comprised disproportionately of women and children.

### Factors that May Increase Risk

Accelerating factors in this model constitute factors that tend to enhance the spread of HIV by worsening dimensions of population vulnerability or increasing HIV exposure opportunity.

#### Factor 1: Increased interaction among military and civilians

Conflict has been demonstrated to result in the increased sexual mixing of military with civilian groups, especially in areas of high military concentration for extended periods of time [25]. In post-conflict settings, demobilization of combatants may also result in disassortive mixing. It has been documented that in the African context, some military groups have higher HIV risk than the general population [26], although this has not been demonstrated conclusively in all contexts. Although military seroprevalence data are not typically available in the public domain, a recent analysis suggests that seroprevalence levels are commonly at least 5% higher among military than their civilian counterparts in Africa [18]. It is important to note, however, that military infection levels vary greatly between and within military organizations. In Cambodia, for example, household surveys estimate prevalence rates of police and military personnel at 8%, while the rate among the civilian population is 2.7% [27,12]. Military recruits in Myanmar, however, appear to have prevalence rates similar to that of the general public [27]. In a large HIV prevalence study in Ethiopia, prevalence among urban military was 7.2%, while among urban civilians the level was 6.4%. Rural military registered a lower rate than the general rural population [28].

#### Factor 2: Increased levels of commercial or casual sex

Impoverishment coupled with discrimination against women and the erosion of traditional behavioral norms may give rise to high levels of sex driven by economic motives in conflict and transitional societies. This again is frequently cited as an important consequence of war, but is difficult to quantify. What is clear is that African countries that have experienced war have lasting socioeconomic effects. For example, Mozambique and Angola are among the three lowest ranking countries on all components of the Human Development Index among Southern African countries [29]. Infant mortality in these two countries rank among the highest in the world. Among refugee and displaced populations poverty results from the severing of livelihood strategies and catastrophic asset loss. Semi-permanent refugee populations that relocate near population centers may be forced to participate in commercial sex in order to ensure household livelihoods or, in extreme cases (e.g. poor female-headed households), to exchange sex for food and other assets [30-32]. In addition, high levels of female illiteracy affect the ability of women to seek alternative forms of livelihood. In northern Uganda, many separated and widowed female Sudanese refugees began brewing and selling beer to sustain themselves and their children. This unfortunately led to an "increase in unprotected sex with multiple partners while under the influence of alcohol" [33]. These vulnerabilities characterize populations in both conflict and post-conflict phases.

#### Factor 3: Decreased availability of reproductive health and other health services

Destruction of the health sector is a common feature of conflicts and its reconstruction has not been rapid in most SSA countries. Massive loss of health infrastructure – both personnel and physical infrastructure – is common in conflict-affected countries of Africa. Mozambique lost the majority of its clinics during the civil war [34]. Rwanda was estimated to have lost more than 80% of its health personnel through death or flight during the genocide [35]. Access and utilization failures result in lack of treatment for STIs and, subsequently, poor health, which may result in greater population-level HIV risk. In Guinea-Bissau, for example, interruption of TB treatment for HIV patients due to the civil conflict was directly responsible for the significantly higher mortality rate experienced by HIV patients. Those who had completed TB treatment before the outbreak of the war, showed no differential increase in mortality [21]. Lack of infrastructure and changing utilization patterns also handicap surveillance efforts [9]. This may delay recognition of HIV, as well as other diseases, as a public health problem.

#### Factor 4: Decreased utilization of reproductive health and other health services

Not only is infrastructure lost, but utilization patterns also are affected by violent conflict. People may not be able to get to health care services because of ambient danger. They may also be reluctant to use services because they distrust providers. This may have been a factor in Rwanda, for example, where providers were particularly implicated in genocide activities during the war [36]. Another deterrent to use is the tendency for conflict-affected populations to become more reliant on self-care, traditional health systems, or emergent predatory health providers, as has been documented in several instances [37].

#### Factor 5: Increased levels of malnutrition

While quality evidence-based research demonstrating direct links between malnutrition and increased susceptibility to HIV seroconversion is scarce, the importance nutrition plays in limiting infection and regulating the immune system is well documented. Malnutrition and micronutrient deficiencies impair immune function, thereby increasing vulnerability to infections in general. Vitamin A, for example, has been shown to play an important role in immune function, including the maintenance of mucosal epithelia, the growth of immunogenic cells and antibody response [38]. Supplementation with vitamin A increases levels of natural killer cells in HIV infected children [39] and decreases morbidity due to other diseases such as measles and malaria [40,41]. Similarly, vitamin E is associated with neutrophil phagocytosis and lymphocyte proliferation [42]. Such deficiencies are associated with accelerated disease progression in HIV patients [43], greater risk of vertical transmission of HIV [44,38] and increased HIV loads [45,46]. It should be noted, however, that other studies have concluded that vitamin A deficiency is not associated with increased vertical transmission [48-52].

In addition, malnutrition levels are generally a good indicator of health status and social equity. High levels of micronutrient deficiency and general malnutrition are widely documented in conflict-affected populations, resulting in decreased resilience to infections. It is known that poor nutritional status is associated with vulnerability to progression from tuberculosis infection to disease. The immunosuppression associated with HIV infection is a major risk factor for the progression of latent tuberculosis infection to active disease and death [53]. The increased level of HIV in a community will certainly contribute to a high dual HIV-TB burden.

#### Factor 6: Decreased use of means to prevent HIV transmission

The isolation and poverty caused by war often results in decreased knowledge of, and access to, means to prevent HIV transmission. Population probability surveys available through the Demographic and Health Surveys, UNICEF's Multiple Indicator Surveys, and specialized HIV behavioral surveys show that knowledge levels and condom use are quite low in conflict-affected countries [9] and that they contrast with regional and sub-regional norms, especially in cases of protracted and widespread conflict. Depressed knowledge levels reflect the failure of mass media campaigns, formal education, and literacy and clinic-based education activities during and following conflict. As illustrated in Table 2, general awareness of HIV as a health threat often significantly surpasses in prevalence the awareness of specific measures that may be taken to prevent transmission, such as the utilization of condoms and the avoidance of multiple sexual partners.

**Table 2:** Knowledge, Attitudes and Practices Related to HIV/AIDS, Selected Countries in SSA

**Country**	**% heard of HIV**	**% know no ways to prevent**	**% know condom use**
Mozambique (DHS, 1997)	82.2	65.8	15.4
Eritrea (DHS, 1995)	80.6	24.2	34.6
Ethiopia (DHS, 2000)	84.7	31.5	33.5
Sierra Leone (MICS, 2000)	54.0	--	27.0
Somalia (MICS, 2000)	36.6	88.3	2.8

Sources: Marco-International, Demographic and Health Surveys (DHS) . UNICEF, Multiple Indicator Cluster Survey (MICS) .

Additionally, knowledge levels tend to exhibit marked intra-national variability, particularly among socially and economically marginalized groups. This variability may be enhanced in conflict-affected countries through regional isolation [54].

The case of Rwanda suggests that conflict may affect contraceptive use and desired family size. Popular wisdom suggests that post-conflict populations may want to repopulate and, to an extent, that tendency is supported by examination of Demographic and Health Survey (DHS) data collected before and after the genocide of 1994. In 1992 and 2000, the DHS documented rates of awareness of HIV exceeding 80% and awareness of modern methods of contraception exceeding 90% [20,55]. Despite the relatively high levels of exposure to information, condom use remained low in 2000, though use is significant in higher risk groups. While this may be due to the disruption of social marketing campaigns and limited access to condoms, desired fertility may have increased since the war, thereby reducing the utilization of modern methods of contraception. From 1992 to 2000, women's rates of utilization of modern methods of contraception decreased from 8.6% to 2.7% (12.9% to 4.3% for women "in union"). During the same period, the percentage of women reporting a desire to have six or more children increased from 14.9% to 28.6% [20,55]. These data highlight the fact that the constraints to adoption of condom use remain significant on the population level and may in part represent a desire to repopulate. In these cases, HIV prevention programs will need to be carefully targeted to take into account this underlying dynamic.

#### Factor 7: Increased population mixing following large internal or regional population movements

On the whole, forced migration may increase HIV risk, as forced migration movement tends to be in a rural-to-urban direction, which greatly enhances the possibility of disassortive mixing. In addition, the migration process is often associated with high levels of physical danger and exposure to sexual violence. Where forced migrants remain in highly insecure areas, migration may be associated with frequent assaults by combatants. In addition, migration patterns may be quite fluid, enabling dislocated populations to move back and forth between higher and lower risk areas, thus increasing disassortive mixing. Research suggests that urban rates of casual/commercial sexual activity tend to exceed rural rates and that exchange of rural/urban populations tends to undermine traditional norms governing sexual activity in rural areas [56]. It has been shown that war can increase partner exchange, as relationships generally tend to be shorter-term, thereby increasing the reproductive rate of the disease [57,58]. Due to the positive relationship between frequency of STIs and HIV, frequent partner exchange and its associated increased risk of STI infection, HIV transmission is further exacerbated [59].

Fluid population movements are particularly common during prolonged conflicts in border areas that might give rise to population mixing and then repatriation. In the case of Rwanda, research illustrates that the post-war period has seen a sharp decrease in the HIV prevalence differential between rural and urban women attending antenatal clinic services in Kigali [60]. The trend may be due in large part to migration between rural and urban communities, combined with the high level of sexual violence inflicted on rural women during the genocide.

#### Factor 8: Emergence of norms of sexual predation and violence

Coupled with increased population movement is the frequent emergence of norms of sexual predation and sexual violence within conflict-affected areas. The phenomenon of rape as a war tactic is increasingly being recognized and documented. The widespread infliction of sexual violence upon women during the Rwandan genocide and its aftermath illustrates the extent to which sexual violence may be utilized as an instrument of war. While exact figures are unavailable, it is estimated that between 250,000 and 500,000 cases of rape occurred during the conflict [61,62]. In Liberia, 49% of women surveyed reported at least one act of sexual or physical abuse by either a soldier or a fighter during the civil war [63]. In Sierra-Leone, 9% of those surveyed reported a war-related incident of sexual violence [64]. Similar accounts have been reported across the world including Kosovo, Azerbaijan, Iraq and others.

In cases of HIV resulting from sexual violence, the shame and social stigma attached to rape prevent women from seeking testing or care. It should be noted that in some conflict-affected populations, research suggests discriminatory cultural attitudes and practices. In Rwanda, research in 1990 found that men control sexual decision-making, and that HIV positive women were more likely to report coercive sex and violence with their sexual partner than HIV negative women [65]. In Angola, of the 38% of women who had been physically abused, 69% had been abused by their husband or boyfriend and 23% by their mother or father [66]. Therefore, HIV treatment and prevention programs need to address the sociocultural determinants of unequal power sharing in sexual partnerships. It should also be noted that in some African cultures including Rwanda, mourning rituals involve intercourse between the widow or widower and another individual. Widows may be forced to have intercourse with a close male relative (i.e. brother or cousin) of her deceased husband to achieve the purification intended from the ceremony [67]. This ceremony, which may occur more frequently during periods of elevated mortality in conflict situations, places the economically vulnerable widow at an elevated risk of HIV transmission.

#### Factor 9: Fragmentation of families and resultant vulnerable household structures

Like HIV, conflict decimates family structures through mortality and dislocation, and results in lasting effects on society [1]. Conflict-affected populations typically have higher rates of child-headed households (e.g. orphans). They may also have higher dependency ratios because of greater numbers of female-headed households and, in some cases, higher levels of handicap and fewer able-bodied men. When juxtaposed with proximity to economically remunerative sex, this change in household ecology would favor greater vulnerability.

## Putting it Together at a Regional Level: Social Ecology of HIV and Conflict

The models and mechanisms identified above help to develop an explanatory model built on a foundation of social ecology. A social ecology approach argues for models of explanation that explore the interactions of multiple causes (environmental, social, and biological) working through different mechanisms and at differing levels of scale (individual, household, community etc.) with emphasis on dynamic change over time. The social ecology of the epidemic is defined in terms of sociodemographic, socioeconomic, structural, contextual, biologic and behavioral variables that facilitate or inhibit progression of the epidemic [68-70]. The ecological perspective explains how conflict might impede the progress of the epidemic and it provides clues as to regional strategic factors that may exacerbate risk in the post-conflict setting. While the epidemiologic literature postulates a number of factors that we do not elaborate here (such as comorbidity and religious practice) these factors also are likely to be important. For the sake of illustrative simplicity, we examine four key variables not commonly cited that are more closely linked to the conflict-HIV axis: (1) population density, (2) level and geographic distribution of economic growth, (3) the prevalence of poverty, and (4) the existence of physical infrastructure, particularly transport infrastructure.

One key feature of mapped data is the relative correspondence of population density, road infrastructure, economic productivity, and HIV risk in Southern Africa (see Figures 3a,3b,3c). Central Africa is marked by more clustered populations and sparse road infrastructure, especially in Congo and Angola – those areas that have experienced chronic conflict. Coastal West Africa has high population density and road density but comparatively low HIV seroprevalence. This may be due in part to poor road quality and lack of road connectedness to areas of high HIV risk. Also the economic productivity of western Africa is much lower than that of southern Africa. Therefore, economic migration patterns may not be as intense. As mentioned earlier, other factors, such as the moderating influence of religion and biological co-factors (e.g. circumcision, and STI rates), may also be important. It is also possible that conflict in central Africa may have moderated HIV risk by buffering the west coast of Africa from movement of HIV up the coast from southern Africa.

Population density may directly influence the occurrence, severity, and spread of violent conflict and HIV. Where population density is lower, population mixing associated with conflict may be more sporadic (as opposed to consistent and sustained). Population density paired with excellent road infrastructure may have a synergistic effect on HIV risk. This may, in part, explain the pattern of risk seen in southern and eastern Africa.

Economics and poverty are important drivers. Although higher economic output is associated with higher levels of HIV in general, the poor are increasingly affected for a variety of well-accepted reasons. From this perspective, Southern Africa remains the most problematic with respect to the potential for rapid northward spread of HIV. Angola and Mozambique are characterized by high poverty, economic potential, and in the case of Angola, the potential to be a gateway to West Africa. One factor that may be driving the epidemic is the interaction between poor women and rich men (or men with some income, such as the military and truck drivers) as exemplified by Drain et al. [68] who found that income inequality was independently associated with HIV seroprevalence.

The ecology of HIV and conflict also results in greater micro-level variation in the determinants of HIV risk. Mock and Drapcho [37] showed that regional variability and the design effects (sample variation across clusters/villages) associated with measures of nutrition and mortality were far greater among post-conflict countries surveyed by DHS than are typically found in more stable settings. This is plausible given that conflict does not have a consistent effect in all areas of countries. Also, it would be expected that functioning social systems found in stable settings might have an equalizing effect on health status.

## How has the International Community Responded to the Joint Effects of HIV and Conflict?

Sadly, the international community has compartmentalized its responses to HIV and conflict – as well as its relief, recovery, and development programming instruments – which precludes an integrated and aggressive attack on the epidemic. As a result, very little has been learned in terms of designing and implementing programs to address HIV among conflicted-affected populations, with some exceptions [71].

The current structure of development assistance results in poor availability of resources to address HIV in conflict settings, as these are traditionally the domain of humanitarian assistance. To date, though much lip service is paid to the concept of developmental relief, the bulk of all humanitarian assistance is supply-side delivery of immediate survival commodities and services. This approach continues to dominate even though humanitarian assistance may be provided to conflict-affected population for years and decades, and even when displacement may provide unique opportunities to reach populations that might otherwise be inaccessible.

Similarly, the immediate post-conflict period is typically serviced by transition programs that emphasize demobilization, reinsertion, reintegration and development of the foundations of governance. While these are clearly important considerations, a problem as severe as HIV cannot be compartmentalized as a development problem (i.e. not a humanitarian or transition-period concern). And indeed, problem-focused activities such as HIV prevention might be a motivating cause for the stimulation of civil society groups.

Even internal to the development community, the problem of HIV is not linked to the problem of conflict, but rather these two issues are seen and treated as unrelated concerns. Different strategies and partners are managed by separate bureaucratic units that have rare interactions. Such compartmentalization of both conflict and HIV along humanitarian/development lines and internal to development management has resulted in fragmented approaches to addresses these important and interactive influences on the health development of SSA.

Similarly, the transitional period should be assessed for opportunities to creatively integrate HIV-related interventions into priority program strategies to promote economic recovery and social rehabilitation. Examples of such integrated programs include integrating HIV education into micro-credit or economic development programs and incorporating HIV risk reduction strategies into demobilization and social integration programs. Demobilized ex-combatants might also be incorporated in educational and health extension programs such that they become part of the solution instead of the problem.

Humanitarian assistance provides numerous opportunities for the cross-sectoral integration of effective HIV-directed initiatives, including the opportunity for synergistic amelioration of adverse health outcomes. 

Even though conflicts typically last for years or decades, countries affected by conflict continue to receive assistance, primarily for immediate survival needs, through humanitarian assistance efforts. In post-accord transition programs, the emphasis is on establishing democratic rule, governance, and re-integration. Sequential application of traditional humanitarian and transition interventions ignores the regional context of high HIV risk. Since HIV risk may be lower in conflict settings while vulnerability to HIV is increasing, this programming approach results in a major constraint to timely, prevention-oriented intervention.

## Implications for Policies and Programs

Our analysis suggests that the relationship between conflict and HIV is complex and contextualized; however, important general conclusions can serve as a basis for action. The analysis demonstrates that while vulnerability to HIV is heightened as a result of conflict, exposure opportunities may be significantly reduced. This may lead to important opportunities for interventions to keep HIV prevalence rates low in the affected settings (i.e. to prevent HIV in relatively lower-prevalence countries). More urgently, post-conflict changes in exposure opportunities could result in explosive epidemic waves. The case of Angola is particularly troublesome given its strategic location and economic potential.

Conflict and HIV are inter-related problems that demand a clear strategy and coordinated use of humanitarian and development assets. This means that objectives for addressing these problems should drive the response, and conflict should be viewed as a key determinant of HIV risk. The HIV/AIDS epidemic is a major constraint to development in Africa. Programs and bureaucracies should more clearly align along a consistent vision of HIV prevention and mitigation in both conflict and post-conflict settings.

A portion of the Declaration of Commitment on HIV/AIDS, adopted at the United Nations General Assembly Special Session on HIV/AIDS (UNGASS) on June 27, 2001, articulates strategies and goals to address HIV/AIDS in conflict and disaster-affected regions. It calls for the development and implementation of national strategies that incorporate HIV/AIDS awareness, prevention, care, and treatment elements into programs or actions that respond to emergency situations. The declaration recognizes that populations destabilized by armed conflict, humanitarian emergencies, and natural disasters – including refugees, internally displaced persons and, in particular, women and children – are at increased risk of exposure to HIV infection, and calls for HIV/AIDS components to be factored into international assistance programs where appropriate [72].

As international, regional and national agencies strive to abide by the Declaration of Commitment on HIV/AIDS, the framework provided here will be useful in the identification of determinants of HIV risk. We recommend that factors affecting HIV risk in conflict settings be systematically assessed as a basis for strategic planning to address HIV in conflict. The assessment should include vulnerability profiling, exposure risk assessment, and characterization of mechanisms through which conflict affects HIV risk. It is particularly important to pre-empt disassortive mixing associated with post-conflict improvements in mobility and resettlement. The assessment should be conducted at the different planning levels (i.e. regional, national and sub-national).

Conflict risk assessment should also be a key component so that synergistic programming between conflict and HIV initiatives can be achieved. Examples of these include:

• potentiating civil society organizations around the HIV problem, especially in areas of high prevalence;

• incorporating HIV prevention as a key element in demobilization and reinsertion initiatives, including the possible use of ex-combatants as HIV educators/mobilizers; and

• aggressive and progressive approaches to poverty alleviation and reduction. Poverty is particularly problematic in chronic conflict contexts, even when societies have high development potential (e.g. the DRC and Angola). Aggressive development programs are probably the most central strategy to confronting HIV and conflict. HIV prevention and conflict resolution can be inserted into a number of the specific components of these programs. Targeting women- and child-headed households, and making it economically feasible for families to send youth to school are also of particular importance.

Our analysis stresses the importance of maintaining a global perspective, while at the same time recognizing that micro-planning may be even more important in conflicted-affected settings than in stable settings. This is because local ecologies may be more diverse due to the varying spatial and temporal effects of conflicts. Indeed, most countries that have experienced large-scale conflict have had quiet zones where life was relatively stable, even during the course of large-scale war. These stable areas could absorb major non-emergency initiatives. In post-conflict settings, these micro-level differences persist, while others, such as differing levels of infrastructure destruction, ethnic tensions and pockets of high HIV prevalence, may require highly specific approaches.

These findings also suggest somewhat different approaches to HIV surveillance in conflict-affected settings. First, a more deliberate attempt should be made to support surveillance in countries and areas affected by conflict so that a better evidence base is developed. Unfortunately, a review of the UNAIDS surveillance database revealed that conflict-affected countries have little or no systematic surveillance, despite the existence of relatively stable areas [73]. This may be due to the fact that lab testing and quality control for HIV surveillance is generally centralized. Perhaps a more de-centralized approach would better ensure continued data collected in conflict-setting such that at least some regions of a country would continue to collect data during conflict. Higher micro-level variation and greater social change in the post-conflict setting argues for more finely graded surveillance. Also, surveillance should take into account the differing risk groups resulting from conflict, and the dynamics of exposure opportunity that occur as a result of opening international borders, rapidly evolving trade corridors and improved internal mobility.

Finally, we argue that information strategies, including HIV/conflict risk assessments and surveillance, should be rationally planned and implemented as a basis for intervention planning and program evaluation during the early phases of conflict response. Seroprevalence or other proxy measures of HIV infection must be monitored more deliberately as a part of surveillance. Conflict-HIV vulnerability/risk assessment tools can be developed based on the factors enumerated above. They should be applied periodically to more effectively respond to a dynamic setting. Improved HIV status information together with these planning data should foster a more evidence-based approach to preventing and mitigating HIV in conflict settings. We call for mainstreaming HIV/AIDS prevention and care policies into conflict prevention, peacekeeping operations, humanitarian responses to crises, post conflict reconstruction planning, implementation and evaluation.

## Competing interests

The author(s) declare that they have no competing interests.

## Authors' contributions

Dr. Mock responsible for overall framework and approach for this paper. Drs. Duale and Brown contributed HIV literature synthesis. Ms. Mathys, O'Maonaigh, Abul-Husn and Elliot extracted literature. All authors read and approved the manuscript.

**Table 1:** HIV/AIDS Risk in Selected Sub-Saharan Africa Countries

	**People living with HIV/AIDS**					
	**% Adults 1999,^1 ^2001^2^**	**Women^2 ^15–49, 2001**	**Children^2 ^0–14, 2001**	**Cumulative AIDS Rate, per 1,000 (year)^1^**	**Estimated Num. of Death due to AIDS, 2001^3^**	**Estimated Num. of AIDS Orphans 2001^4^**

**ANGOLA**	2.8, 5.5	190,000	37,000	0.2 (1997)	24,000	104,000
**BOTSWANA**	35.8, 38.8	170,000	28,000	4.7 (1998)	26,000	69,000^3^
**BURKINA FASO**	6.4, 6.5	220,000	61,000	1.0 (1997)	44,000	268,000
**BURUNDI**	11.3, 8.3	190,000	55,000	1.7 (1996)	40,000	237,000
**CONGO**	6.4, 7.2	59,000	15,000	3.9 (1998)	11,000	78,000
**CONGO DR**	5.1, 4.9	670,000	170,000	0.8 (1998)	120,000	927,000
**COTE D' IVORIE**	10.8, 9.7	400,000	84,000	2.6 (1996)	75,000	420,000
**ERITREA**	2.9, 2.8	30,000	4,000	1.3 (1998)	350	24,000
**ETHIOPIA**	10.6, 6.4	1,100,000	230,000	1.3 (2000)	160,000	989,000
**GUINEA**	1.5 (1999)	29,000^5^	2,700^5^	0.6 (1998)	5,600	29,000
**KENYA**	14, 15.0	1,400,000	220,000	2.7 (1998)	190,000	892,000
**LIBERIA**	2.8 (1999)	NA	2,000	0.1 (1998)	4,500	39,000
**MOZAMBIQUE**	13.2, 13.0	630,000	80,000	0.6 (1998)	60,000	418,000
**NAMIBIA**	19.5, 22.5	110,000	30,000	4.1 (1997)	13,000	47,000^3^
**NIGERIA**	5.1, 5.8	1,700,000	270,000	0.2 (1999)	170,000	995,000
**RWANDA**	11.2, 8.9	250,000	65,000^3^	2.2 (1997)	49,000	264,000
**SIERRA LEONE**	3.0, 7.0	90,000	16,000	0.0 (1996)	11,000	42,000
**SOUTH AFRICA**	19.9, 20.1	2,700,000	250,000	0.3 (1996)	360,000	660,000^3^
**TANZANIA**	8.1, 7.8	750,000	170,000	3.2 (1998)	140,000	815,000
**UGANDA**	8.3, 5.0	280,000	110,000	2.5 (1997)	84,000	884,000
**ZAMBIA**	20, 21.5	590,000	150,000	5.0 (1997)	120,000	572,000

^1 ^International Program Center, Population Division, U.S. Census Bureau. Updated June 2000. HIV/AIDS Surveillance Data Base. "HIV /AIDS Country Profiles." Online  [accessed April 5, 2002].

^2 ^United Nations Development Program. 2002. "Human Development Indicators 2002." Aggregates calculated for the Human Development Report Office by UNAIDS. Online  [accessed January 14, 2003].

^3 ^UNAIDS, WHO. Updated 2002. "Epidemiological Fact Sheets on HIV and Sexually Transmitted Infections."  [accessed January 22, 2003].

^4 ^Joint USAID/UNICEF/UNAIDS Report. 2002. "Children on the Brink." Appendix I. Online  [accessed January 14, 2003].

^5 ^United Nations Development Program. 2001. "Human Development Indicators 2001." Aggregates calculated for the Human Development Report Office by UNAIDS. Online  [accessed January 14, 2003].
